# Surgical Approaches in Total Hip Arthroplasty

**DOI:** 10.26502/josm.511500106

**Published:** 2023-05-15

**Authors:** Rajiv Supra, Rajesh Supra, Devendra K Agrawal

**Affiliations:** 1 College of Osteopathic Medicine, Touro University, Henderson, Nevada, USA; 2 Georgetown University School of Medicine, Washington, DC, USA; 3 Department of Translational Research, College of Osteopathic Medicine of the Pacific, Pomona, California, USA

**Keywords:** Anterolateral approach, Direct anterior approach, Direct lateral approach, Periprosthetic joint infection, Posterior approach, THA dislocation, Total hip arthroplasty

## Abstract

The Total Hip Arthroplasty (THA) has become one of the most reliable surgical interventions that has improved the quality of life in many patients. THA allows patients to have increased mobility, range of motion, and reduced pain in patients with degenerative hip joints. This surgical procedure has become an effective treatment option for several chronic conditions affecting the hip joint. Although this surgery has been shown to give promising results in patients with hip pathology, selecting the approach for THA is a critical step in pre-operative planning. The best approach for this surgical procedure depends on multiple factors and each present with their own challenges, success rates, and limitations. To further elucidate the advantages and disadvantages associated with different surgical approaches, we critically review each surgical approach along with the different causes of failure of the THA procedure.

## 1. Introduction

Total hip arthroplasty (THA) is a highly effective surgery for reducing pain and improving the quality of life in patients with hip osteoarthritis [1]. THA has been researched overtime and shown to have 10-year survivorship in more than 95% cases [2]. Over one million THA are performed every year and is projected to reach two million procedures a year by the year 2030 [1].

**Figure 1:**
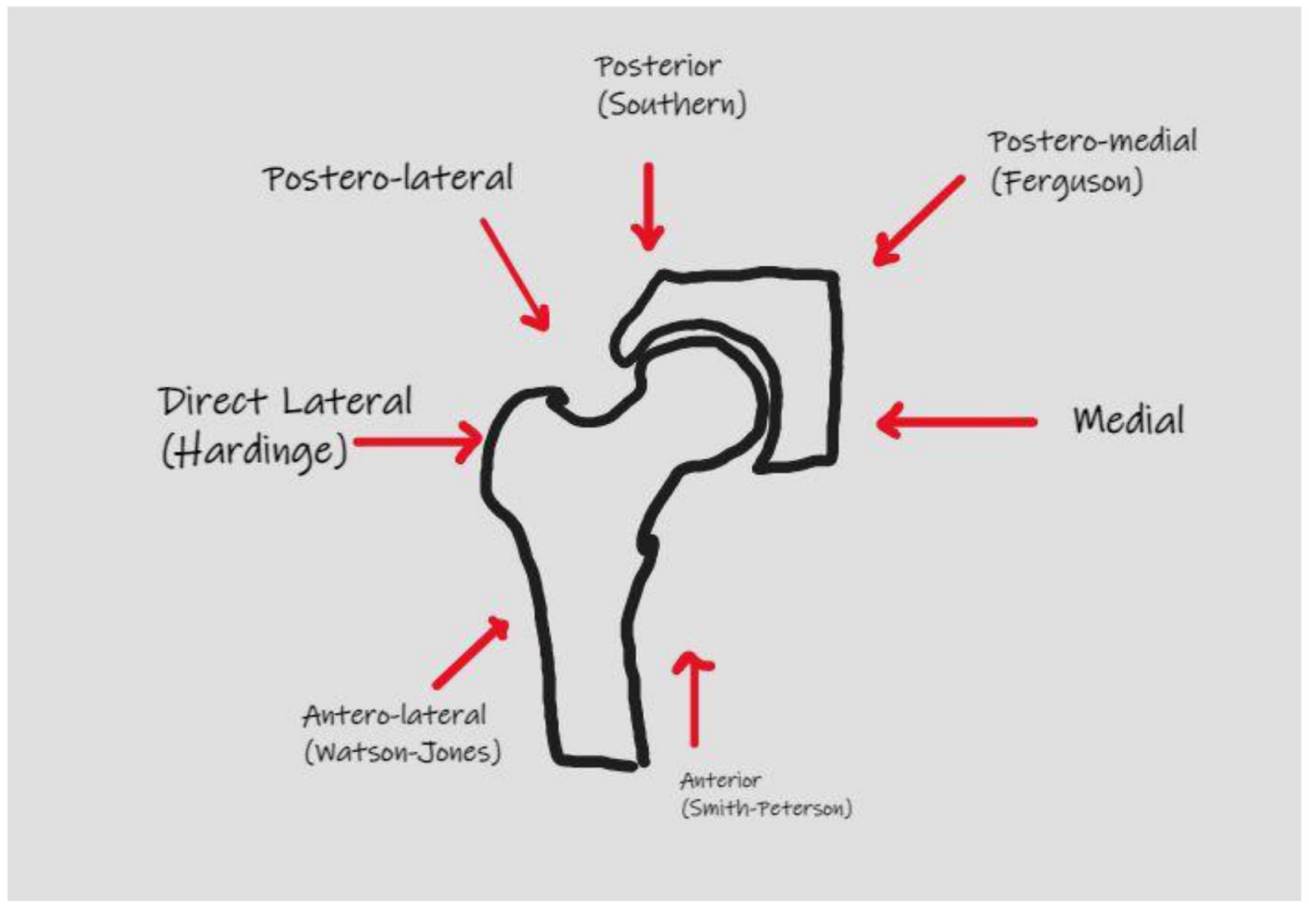
Surgical approaches to Total Hip Arthroplasty.

There are many surgical approaches when conducting this procedure such as the Direct Anterior Approach (DAA), Posterior Approach (PA), and Lateral Approach (LA), all of which have their respective advantages and pitfalls (Figure 1). The PA approach involves splitting the gluteus maximus muscle which allows exposure of the femur and acetabulum, avoiding disruption of the hip abductors all together [3]. However, this approach has an increased rate of dislocation when compared to the DAA or LA [4]. The LA begins with splitting the gluteus medius muscle to access the hip anterolaterally. This surgical approach has been documented to have the lowest risk of dislocation but is associated with superior gluteal nerve injury, impaired abductor function, and heterotopic ossification [3]. The DAA is different with its intermuscular and inter-nervous plane between the tensor facia latae and sartorius. Advantages with DAA is earlier recovery, lower dislocation rates, and shorter hospital stays [3]. Disadvantages include increased risk of periprosthetic fractures, increased learning curve, and higher risk of injuring the Lateral Femoral Cutaneous Nerve (LFCN) [5]. This article highlights the techniques for each surgical approach and a critical review of outcomes and complications.

## 2. Approaches in Total Hip Arthroplasty

### Direct Anterior Approach (DAA):

The DAA enters the hip through an intermuscular plane between the tensor fasciae latae and gluteus medius laterally and medially through the sartorius muscle and rectus fascia [6]. The DAA is considered soft tissue sparing as it preserves the stability of the hip joint when compared to the posterior approach. Patients have been documented to experience a rapid recovery based on function and activity using the DAA making this approach potentially advantageous when compared with other approaches [6,7]. Additionally postoperative narcotic use is considerably lower in DDA compared to other surgical approaches [8]. The length of stay has been found to be shorter and more patients are being discharged home as opposed to transfer to post op care facilities [9]. Motor function recovery is faster and the time for ambulation without the use of assisted devices is also shorter. The average time to discontinue the use of walkers or canes was 21 days [10].

The DAA spares abductor muscles and the accuracy of the acetabular cup positioning has been shown to be better with less variation in cup angle when compared to the posterior approach [11,12]. Although there is a tendency for the insertion of the acetabular cup in an anteverted fashion, revision rates for acetabular cup failure were lower in DAA compared to the posterior approach [13]. Nerve injuries are also a risk with DAA. The LFCN has been reported to be among the most injured nerve with the DAA. The LFCN arises from the second and third lumbar nerves and is purely sensory. It courses along the psoas major muscle, crosses the iliacus, and runs below the anterior superior iliac spine [14].

The most important predisposing factor for LFCN injury is the branching pattern. A study described 4 major branching patterns identified as trifurcate, primary femoral, late, and classical. The late branch was found to be at highest risk for injury due to its perpendicular orientation to the incision line [15]. The branching pattern is difficult to predict prior to surgery and the best way to avoid nerve damage is to be cognizant of the various anatomical variations. The rate of injury of the LFCN associated with the DAA is variable between studies with reports ranging from 0.1% to 81% [16,17]. Fortunately, injury to the LFCN does not lead to major neurological deficits and patients report sensations of numbness or burning in the anterolateral region of the high at worst [18].

Early rates of THA failure have been reportedly increasing ranging from 24% to 50% within 5 years of the surgery due to femoral or acetabular prosthetic fracture, mal-alignment, or instability [19-21]. A study reported early femoral periprosthetic fractures and loosening were significantly higher in the DAA when compared to the LA and PA. Additionally, early femoral component loosening was higher with DAA than LA and PA [19,22]. Malalignment of the acetabular cup causes impingement, dislocation, and loosening of the acetabular component [23]. Femoral stem alignment and acetabular anteversion may differ among approaches but has been shown to be more difficult to implant the femoral stem in a neutral position through the anterolateral approach. This was due to elevating the proximal aspect of the femur in the anterolateral surgical approach which may also be true for DAA [20,24]. The tendency to insert the acetabular cup in a more anteverted orientation is seen with DAA and femoral stem anteversion may be mitigated by using the posterolateral approach [25].

### Posterior Approach

The inception of the PA dates to 1874 with Bernhard Langenbeck who used this technique at the time to treat arthritis of the hip joint [26]. Later this technique was modified by detaching the short external rotator tendons and the gluteus maximus tendon from the femur to better visualize the hip joint [27]. PA has been reportedly the most used surgical approach for THA [28]. This surgical technique is performed by positioning the patient in lateral decubitus position. The pelvis is stabilized with a padded board placed anterior to the pubic symphysis and chest as well as posterior to the shoulder blades. Additionally, a padded roll should be placed under the contralateral chest wall to reduce incidence of brachial plexopathy. Incision begins 5 cm distal to the greater trochanter and near the center of the femoral diaphysis. The incision continues down and curves toward the superior iliac spine where the skin and subcutaneous fat are separated down to the fascia lata and Iliotibial Band (ITB). The ITB and fascia lata are separated longitudinally to split along the gluteus maximus. Retractors are then placed to split the gluteus maximus and better visualize the piriformis and short external rotators (SERs). The SER are then separated from the greater trochanter and reflected posteriorly to better visualize the posterior hip capsule [29].

### Risk of dislocation

It has been noted, historically, that the PA has been associated with greater dislocation rates when compared to the LA. Studies revealed the occurrence of almost 10% dislocation rate after THA using PA compared to the anterolateral approach [30]. Similarly, a review of several studies examining over 10,000 THAs revealed the posterior approach group had a 6-fold higher dislocation rate than LA [29]. A critical factor of the PA was the enhanced soft tissue capsule repair at the end of the surgical procedure which helped diminish dislocation events [31]. A meta-analysis by Zhou et al. [32] revealed THAs with soft tissue capsular repair had lower dislocation rates than those without capsular repair. Kwon et al. [33] however, demonstrated that PA done with repair had higher rates of dislocation when compared to the LA. A prospective study by Meneghini et al. [34] showed that higher dislocation risk was not significantly associated with the PA Interestingly, a cohort study by Fessy et al. [35] revealed PA alone was not a risk factor for dislocation when compared to DAA and LA. Additionally, a randomized controlled trial compared DAA and PA regarding muscle damage. This trial measured inflammatory markers on post-operative days 1 to 4. Investigators concluded the DAA had less muscle damage as measured by inflammatory markers. However, no functional difference between the 2 groups were noticed when looking at 6-month Harris hip scores [35]. While the literature indicates different surgical approaches have variable muscle damage, most studies reveal equal outcomes around 6 weeks postoperatively and further research is warranted in this field [36].

### Lateral Approach

The first description of a lateral approach to THA was described as dissecting the interval between the gluteus minimus and medius tendons as well as the vastus lateralis [37]. This approach exposed the femoral head and in 1954, Osborne described a variation that involved placing the patient in a lateral decubitus position. This method involved removing the gluteus medius tendon from its attachment off the trochanter and conducting a subperiosteal dissection from the trochanter continuous with the vastus lateralis tendon [29,38]. The most well-known LA was described by Hardinge and involved splitting the gluteus medius and minimus tendons longitudinally and continuing distally into the vastus lateralis tendon [39]. Soon after, the anterolateral approach gained popularity and was well developed by Sir Watson-Jones. This approach involved the interval between the tensor fasciae latae and the gluteus medius tendon. The gluteus medius tendon was removed to allow for anterior dislocation of the hip joint [40]. Several modifications were created over the decades for the LA. Modifications were made such as removing the abductor muscles and repairing them in different variations. The Rottinger approach modified the LA by sparing detaching the abductor before exposing the joint capsule [41,42].

The LA also is now used in a minimally invasion approach. In the last decade, abductor sparing approaches have been used with early recovery in outpatient total hip programs. As up to 99% have been successfully discharged on the day of the THA using the minimally invasive anterolateral approach [43,44]. A randomized controlled trial compared 40 patients undergoing LA with patients using the DAA. The study examined several outcomes including the gait speed test, timed up and go test and concluded the DAA had better initial functional recovery up to 2 weeks post op. However, no difference was seen with respect to operative blood loss, procedure time, or length of stay in the hospital [45]. Furthermore, a study examined the LA and DAA while measuring damage with preop and postop lab values, muscle atrophy using MRI, and hip outcome scores. All preoperative demographics were equal amongst patients and lab values used to measure muscle damage included erythrocyte sedimentation rate, C-Reactive Protein (CRP), and acute inflammatory cytokines. Investigators revealed creatine kinase and CRP were higher in LA on postop day 4 and all lab values equalized 1 month post THA. Moreover, MRI 6 months post op showed increased gluteal fatty atrophy in LA [46,47].

### Complications Using Different Approaches to THA

**Figure 2:**
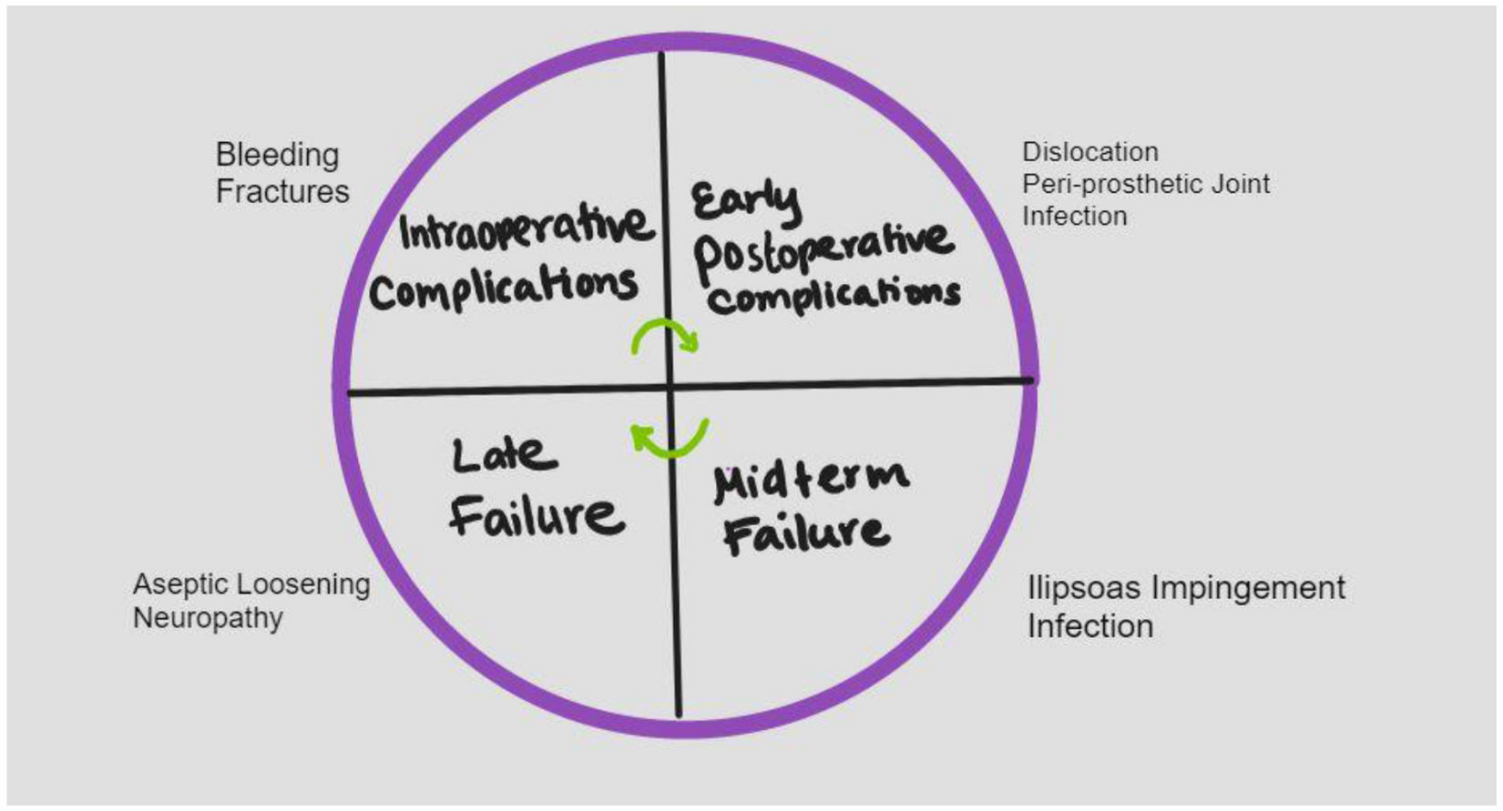
Potential causes of failure and complications following Total Hip Replacement.

Regardless of the approach to performing a THA, post-surgical complications remain an issue for many patients. Advances in implant design, the use of advanced protocols for controlling infections, and the use of minimally invasive procedures have improved outcomes of THA [48]. However, possible causes of THA failure including periprosthetic joint infection, dislocation, iliopsoas impingement, and implant malalignment remain issues that must be addressed to improve outcomes of THA (Figure 2).

### Dislocation

Dislocation has been one of the most common issues after a primary THA. Risk factors have been identified from several studies related to implant and surgical technique used. A meta-analysis revealed obesity as a risk factor associated with a higher risk of dislocation compared with non-obese patients [49]. Spinal fusion has also been shown to increase rates of dislocation [50]. Dislocation occurs when the forces on the implant head overcome resistance from soft tissues helping to prevent travel of the head. Selecting a larger sized head such as a 32 mm or 36 mm head is a simple option that can prevent dislocation [51,52]. Impingement can also occur leading to dislocation when bone-to-bone or implant-to-implant contact each other during hip motion [51]. Impingement results from malposition and residual osteophyte formation [53]. Other factors contributing to dislocation are lumbar fusion, posterior pelvic tilt, and conditions causing vertebral fusion [54,55]. Capsular repair also plays a critical role in dislocation. The posterior approach is associated with a higher risk of dislocation, however, studies revealed that capsular repair reduced the risk of dislocation [56].

### Infection

Periprosthetic Joint Infection (PJI) can occur any time after the operation and is the most troublesome cause of failure after a hip replacement. A recent study of a registry database revealed that a mean 90-day revision rate for infection was 0.3% for THA and a meta-analysis of PJI found the overall incidence was 0.4% [57,58]. Risk factors for PJI include high body mass index, surgical site infection, diabetes mellitus, chronic pulmonary disease, cardiovascular disease, and allogenic blood transfusion [59,60]. Preoperative screening for risk factors is critical to reduce the incidence of infection in THA [61]. Perioperative antibiotic prophylaxis has also been researched to prevent PJI and data supports the use of cephalosporins [62]. Additionally, instead of using normal saline solution lavage, the data suggests povidone-iodine lavage for intraoperative irrigation [63]. Debridement and antibiotics for PJI in well-fixed implants is the first option for treatment. Critical factors for the success of this option involves early intervention, typically within seven days of onset, and replacing all modular components [64]. A recent meta-analysis revealed the use of rifampin in successfully treating PJI caused by any species of Cutibacterium, Streptococcus, and Staphylococcus. Results revealed combined use of agents with rifampin posed better outcomes than not using rifampin [65].

### Nerve Impingement

Iliopsoas impingement causes pain upon active hip flexion and is a complication of THA [66]. A study reported the diagnosis of iliopsoas tendonitis was made at roughly 2.8 months post THA [67]. Symptoms of iliopsoas impingement appear after full recovery and do not appear immediately after surgery. Female sex increased acetabular to femoral head diameter, and female sex have been researched to be contributing factors for iliopsoas impingement [68]. In addition to iliopsoas tendonitis, the type of implant used for THA has been associated with higher rates of failure. For example, higher rates of failure were reported after use of large head and metal on metal bearings [69]. Furthermore, a severe post-op complication after THA is nerve palsy. Several nerves have been documented that can be potentially damaged during THA including the femoral and sciatic nerve [70]. Risk factors for nerve damage include revision arthroplasty, female sex, dysplastic osteoarthritis, and limb elongation [71]. Nerve damage leads to motor paralysis affecting patient activity and overall wellbeing post THA. Recovery of femoral nerve palsy has been shown to be more predictable than sciatic nerve injury [71].

### Fractures

Other common post-operative complications after a THA include pain and periprosthetic fractures. A systematic review of 24 studies of postop pain after THA revealed causes of pain such as adhesions, trochanteric bursitis, heterotopic ossification, capsular fibrosis, and neuropathic pain. Of these, heterotopic ossification and bursitis were among the easiest to diagnose using imaging techniques and can be easily resolved through surgical management [72]. Additionally, periprosthetic fracture remains one of the most troublesome consequences post THA in older patients with osteoporosis [73]. This condition presents at any stage during a THA from intraoperatively to years after the surgery. A meta-analysis studying risk factors for periprosthetic factures revealed age greater than 80 and female sex as well as rheumatoid arthritis being most associated with post-operative fractures [74]. Risk assessment tools such as FRAX scores have been used to identify the risk of fractures post THA related to osteoporosis [75]. Several anti-osteoporosis drugs have been identified and used to prevent bone loss and fractures post THA, yet the clinical importance of such therapeutics has yet to be verified in an aging population [76,77].

### Bone and Blood Loss

Extensive bleeding occurs during THA and in many cases requires blood transfusions during surgery which carries a risk of increased morbidity and mortality. A meta-analysis researched the effects of blood loss during THA and compared the DAA to the LA. The DAA was found to have less blood loss due to the intramuscular and inter-nervous approach [78]. Another study revealed the anterolateral approach had lowest rates of blood loss compared to the posterolateral approach which showed highest rates of blood loss [79]. These results were congruent with a separate study that showed the anterolateral approach having lower estimated levels of blood loss [80].

Bone loss is another potential complication following THA and must be considered when deciding an approach. Studies reveal that bone loss occurs at the proximal femur and greater trochanter which increases fracture rates around the implant. One study investigated the effects of using an anterolateral approach and direct lateral on periprosthetic bone loss in patients with femoral neck fracture. Bone loss was measured using bone mineral density and the study reported that at the 3 month follow up, higher rates of bone loss occurred in the patients using the direct lateral approach which subsequently increased at 6 months. Hence, surgical approach does have an influence on periprosthetic bone loss and surgical approaches must be considered to minimize bone loss [81].

### Trendelenburg Sign

When a patient has weak hip abductors, the Trendelenburg sign can be observed. Different surgical approaches used in THA results in either the gluteus medius being split, retracted, or spared as mentioned above. The Trendelenburg gait is a well-known residual effect post THA, and the surgeons should choose an approach where these muscles are minimally affected. In a randomized controlled trial studying the DAA and the LA, patients who underwent the LA showed a positive Trendelenburg sign at higher rates [82]. If patients have not regained muscle strength at the 24-month mark, studies revealed they are unlikely to regain normal muscle strength. This was supported by other randomized controlled trials comparing the anterolateral and the LA which both showed high rates of Trendelenburg sign [83]. In a 6 year follow up study comparing PA and LA, the Trendelenburg sign was positive in most of the patients who undergone the LA whereas no signs were seen in those who were operated on using the PA [84]. Additionally, a systematic review revealed that the Trendelenburg sign was commonly seen with the LA than with the PA. Reinsertion, splitting, or retracting the gluteus medius can result in a positive Trendelenburg gait following THA. In the mentioned studies, the LA had higher rates of abnormal gait and the surgeon must be cautious when using this approach [85].

## 3. Conclusion

Total hip arthroplasty can be done using a wide range of surgical approaches. The DAA, LA, and PA are among the most common. Each approach comes with its own unique advantages and disadvantages. Even though the PA is the most common approach, all other methods fall short when considering infection rates, bone loss, and accessibility. Therefore, the surgeon should take caution when selecting a surgical approach.
